# Novel fermented chickpea milk with enhanced level of *γ*-aminobutyric acid and neuroprotective effect on PC12 cells

**DOI:** 10.7717/peerj.2292

**Published:** 2016-08-04

**Authors:** Wen Li, Mingming Wei, Junjun Wu, Xin Rui, Mingsheng Dong

**Affiliations:** 1 College of Food Science and Technology, Nanjing Agricultural University, Nanjing, PR China; 2 Jiangsu Key Construction Laboratory of Food Resource Development and Quality Safe, Xuzhou Institute of Technology, Xuzhou, PR China

**Keywords:** Chickpea, γ-aminobutyric acid (GABA), *Lactobacillus plantarum*, Neuroprotective effect

## Abstract

In this study, novel fermented chickpea milk with high *γ* -aminobutyric acid (GABA) content and potential neuroprotective activity was developed. Fermentation starter that can produce GABA was selected from 377 strains of lactic acid bacteria isolated from traditional Chinese fermented foods. Among the screened strains, strain M-6 showed the highest GABA-producing capacity in De Man–Rogosa and Sharp (MRS) broth and chickpea milk. M-6 was identified as *Lactobacillus plantarum* based on Gram staining, API carbohydrate fermentation pattern testing, and 16s rDNA sequencing. The complete gene encoding glutamate decarboxylase was cloned to confirm the presence of the gene in *L. plantarum* M-6. The fermentation condition was optimized by response surface methodology. Results demonstrated that * L. plantarum* M-6 produced the highest GABA content of 537.23 mg/L. The optimal condition included an inoculum concentration of 7%, presence of 0.2% (m/v) monosodium glutamate and 55 µ M pyridoxal-5-phosphate, incubation temperature of 39 °C and fermentation time of 48 h . GABA-enriched chickpea milk exerted protective effects on PC12 cells against MnCl_2_ -induced injury. GABA-enriched chickpea milk improved cell viability and markedly attenuated the release of lactate dehydrogenase compared with the impaired cells.

## Introduction

Gamma-aminobutyric acid (GABA) is a non-protein amino acid that is naturally present in microorganisms, plants, and animals. GABA acts as the major inhibitory neurotransmitter in the mammalian central nervous system (Li & Cao, 2010). This amino acid also exhibits several physiological functions, such as inducing insulin secretion (Adeghate & Ponery, 2002), regulating the rate of protein synthesis in the brain (Tujioka et al., 2009), and reducing blood pressure (Inoue et al., 2003). GABA deficiency in the brain is related to neurological disorders, such as Parkinson’s and Huntington’s diseases (Cho, Chang & Chang, 2007). In addition, GABA can improve the function of visual cortex cells and thus can be used to alleviate decline in sensory, motor, and cognitive skills, which occur with aging (Leventhal et al., 2003). In this regard, researchers have focused on developing functional foods containing GABA. However, direct addition of GABA to food is considered unsafe and unnatural (Seok et al., 2008). Utilization of GABA-rich foods produced by natural techniques, such as fermentation, is thus preferred.

Lactic acid bacteria (LAB) are widely used in fermented food and generally regarded as safe (Molina et al., 2012). Some LAB strains can produce GABA by catalyzing the *α*-decarboxylation of L-glutamic acid or monosodium glutamate (MSG) via the enzyme glutamate decarboxylase (GAD); this enzyme is dependent on pyridoxal 5′-phosphate (PLP) or vitamin B6 (Diana et al., 2014). GABA is generated by LAB during fermentation of different kinds of food, such as bovine milk (Inoue et al., 2003), black raspberry juice (Kim et al., 2009), and black soybean milk (Ko, Lin & Tsai, 2013). Chickpea, the third most important pulse crop, is extensively produced in several regions worldwide (Roy, Boye & Simpson, 2010). Chickpea is rich in proteins, vitamins, and glutathione and low in fat (Xiao et al., 2014); this legume is also used in Uygur folk medicine in China. Chickpea is a good source of *α*-galactooligosaccharides (5%–10%) (Xiang et al., 2008); these polysaccharides facilitate the growth of LAB and are suitable for GABA production.

This work aims to develop GABA-rich chickpea milk through fermentation using an LAB strain with high capacity to produce GABA. Various LAB strains were screened based on their capacity to produce GABA. The fermentation conditions of the chickpea milk were then optimized. The protective effects of fermented chickpea milk extracts (FCE) were also assessed based on MnCl_2_-induced toxicity in PC12 cells to illustrate the functional properties of the fermented product.

## Materials and Methods

### Materials

Chickpeas (Kabuli) were purchased from a supermarket in Xinjiang Uygur Autonomous Region, China. The rat pheochromocytoma cell line (PC12 cells) was purchased from KeyGEN BioTECH (Nanjing, China). GABA, 3-(4, 5-Dimethylthiazol-2-yl)-2,5-diphenyl tetrazolium bromide (MTT) and the solvents used for HPLC were acquired from Sigma–Aldrich Chemical Co. (St. Louis, MO, USA). Dulbecco’s modified Eagle’s medium (DMEM) and fetal bovine serum (FBS) were supplied by Gibco Ltd. (Grand Island, NY, USA). All other chemicals used were of analytical grade and purchased from Sinopharm Chemical Reagent Co. Ltd. (Shanghai, China).

### Bacterial strains and culture conditions

LAB strains with high GABA production capacity were screened from 377 strains isolated from traditional fermented food. LAB was activated for two successive transfers in De Man–Rogosa and Sharp (MRS) broth (Merck, Darmstadt, Germany) at 37 °C for 14–16 h prior to use; the strain was then cultured in MRS containing 1% (w/v) MSG at 37 °C for 48 h.

### GABA assay using pre-staining paper chromatography

GABA levels were determined qualitatively using the method described by Li et al. (2009) with slight modifications. The culture broth was centrifuged at 10,000 ×*g* for 10 min, and 1 µL of the supernatant was spotted onto chromatography paper. The paper was developed using n-butanol–acetic acid–water (2:1:1) containing 0.8% ninhydrin. The paper was then dried in a convection oven at 80 °C for 30 min for color development.

### GABA and MSG assay by high-performance liquid chromatography (HPLC)

GABA and MSG in the supernatant were determined by HPLC using the method of Ko, Lin & Tsai (2013) with minor modifications. HPLC analysis was performed with a ZORBAX Eclipse Plus C18 reversed-phase analytical column (4.60 mm × 250 mm, 5 µm, Agilent). Briefly, 100 µL of the sample was added into 500 µL of boric acid buffer solution (0.4 M, pH 10.4) and 100 µL of derivatization reagent (containing 10 mg of *ortho*-phthalaldehyde (OPA) and 10 µL of 2-mercaptoethanol in 2.5 mL of acetonitrile). The mixture was vortexed for 30 s and reacted at room temperature for 5 min. Each sample (20 µL) was injected and monitored at 334 nm wavelength by using a DAD detector (G1315 B). The elution solvent system consisted of (A) 0.02 M ammonium acetate buffer (pH 7.3) and (B) HPLC-grade acetonitrile. The program was set at 20% of B for 10 min, ramped at 100% for 20 min, then at 20% of B for 25 min. A flow rate of 0.6 mL/min was used. The temperature of the column oven was set at 25 °C. The calibration curve was obtained based on six levels (100, 200, 300, 400, 500, and 600 mg/L) of the GABA standard.

### Liquid chromatography–mass spectrophotometry (LC–MS)

The molecular weight of the OPA derivative of GABA was determined through LC–MS (Agilent 1200 and 6410 triple quadrupole mass spectrometry series; Agilent, Palo Alto, CA, USA). The Triple Quad mass spectrometer was equipped with an ESI and operated in positive-ion mode. The identification conditions were as follows: HV voltage, 3.5 kV; capillary, 4 nA; nebulizer, 30 psi; gas temperature, 300 °C; gas flow, 10 L/min; and scan range, m/z 50–400 units.

### Preparation of fermented chickpea milk by using LAB

Chickpeas were washed and soaked in distilled water for 12 h at room temperature. Water was decanted, and the soaked chickpeas were boiled with distilled water (w/v) 10 times for 15 min by using an electric pot (C21-SH808, Shandong Joyoung Small Household Electrical Appliance Co., Ltd., Jinan, China). Chickpeas were wet-milled continuously for 5 min by using a homogenizer (BE601AB, Midea, China) and filtered through double-layered cheesecloth. The filtrate of chickpea milk was added with 0.2% MSG and sterilized at 108 °C for 15 min. Fermentation was started by inoculation of LAB to achieve an initial count of 10^7^ cfu/mL, followed by incubation at 37 °C for 48 h. The fermented chickpea milk was stored at 4 °C for 12 h after maturation.

### Sensory analysis

Sensory analysis was performed in accordance with the method of Lawless & Heymann (1999) with minor modifications. Ten trained panelists familiar with basic sensory evaluation skills were asked to assess the appearance, aroma, texture, flavor, taste, and overall acceptability of the fermented chickpea milk by using a five-point scale (1 = dislike very much; 2 = dislike; 3 = acceptable; 4 = like; 5 = like very much).

### Identification of LAB

Carbohydrate fermentation patterns of the strain M-6 were determined using the API 50 CHL kit (bioMérieux, France). The kit contains 50 biochemical experiments for examining the carbohydrate fermentation pattern of LAB strains, thereby enabling the identification of the strain at the species level. The strain was further identified by 16S rDNA sequencing (Chin et al., 2006).

### Cloning of the *L. plantarum* M-6 GAD gene and sequence analysis

Genomic DNA was extracted according to the method of Van der Meulen et al. (2007) and used as template for subsequent experiments. DNA encoding GAD was amplified by using the forward primer 5′-ATGGCAATGTTATACGGTAAACAC-3′ and the reverse primer 5′- TCAGTGTGTGAATAGGTATTTCTTAGGT-3′, which were designed using Primer 5 software based on the GAD sequences collected from NCBI. The reaction program was set as follows: 5 min at 94 °C; followed by 25 cycles of 30 s at 94 °C, 30 s at 55 °C, and 180 s at 68 °C; and an additional extension at 68 °C for 5 min. After agarose gel electrophoresis, the amplified product was purified using BiosPure Gel Extraction Kit (Hangzhou Biosci Biotech Co. Lid, Hangzhou, Zhejiang, China), ligated into pMD19T (simple) by T4 DNA ligase (Thermo Scientific, Waltham, USA), and transformed into *E. coli* JM109. Positive transformants were selected on Luria–Bertani plates containing ampicillin and confirmed through colony PCR; the validated positive clone was verified by sequencing (Sangon Biotech, Shang Hai, China). The GAD sequence obtained in this study was subjected to similarity search in the GenBank database.

### Determination of LAB counts and pH

Viable count was determined using MRS agar plates. An aliquot of 1 mL of the sample was extracted from the flasks and diluted by 10-fold in sterile physiological saline (0.85%, w/v). The diluted sample was inoculated onto MRS agar plates and incubated at 37 °C under 5% CO_2_ for 48 h. Viable colonies were counted and expressed as log (cfu/mL). pH was measured using a pH meter (Lab850, Schott, Germany).

### Response surface methodology (RSM) experimental design

A three-level, three-factor factorial Box–Behnken design (BBD) of response surface methodology (RSM) was performed. Inoculum concentration (A), temperature (B), and PLP (C) were selected as independent variables, and GABA content was chosen as dependent variable. The independent variables were studied at three different levels (−1, 0, + 1), and a set of 17 experiments were performed (Table 1). Design Expert software v7.0.0 (StaEase Corp., Minneapolis, MN, USA) was used to analyze the experimental data.

**Table 1:** Box–Behnken design and responses to GABA yield in fermented chickpea milk.

Trials	A Inoculum concentration (%)	B Temperature (°C)	C PLP (µM)	GABA(mg/L)	
				Predicted	Observed
1	−1(3)	0(37)	−1(20)	325.57	326.54 ± 3.77
2	0(5)	0	0(50)	467.53	495.53 ± 5.11
3	0	1(42)	−1	356.08	346.62 ± 8.48
4	−1	1	0	311.12	319.61 ± 8.02
5	1(7)	0	1(80)	453.05	452.08 ± 2.90
6	0	0	0	467.53	479.85 ± 3.57
7	0	1	1	398.02	395.05 ± 5.27
8	-1	0	1	335.07	329.54 ± 5.11
9	0	0	0	467.53	412.97 ± 7.12
10	0	−1(32)	1	111.39	120.85 ± 1.60
11	0	−1	−1	112.57	115.54 ± 0.87
12	1	0	−1	421.79	427.32 ± 3.50
13	−1	−1	0	108.30	104.36 ± 1.25
14	1	−1	0	153.15	144.65 ± 9.21
15	0	0	0	467.53	479.26 ± 7.75
16	0	0	0	467.53	470.06 ± 4.99
17	1	1	0	480.47	484.41 ± 3.79

### Protective effects of FCE on manganese-induced PC12 cell death

#### Determination of cell viability by MTT assays

MTT assays were used to evaluate the protective effect of FCE on manganese -induced PC12 cell death by measuring cell viability (Lee, Yoon & Park, 2008). PC12 cells were cultured in DMEM containing 10% FBS and 1% penicillin/streptomycin. The cell suspension was seeded into 96-well plates at a concentration of 2 × 10^5^/well and incubated at 37 °C for 24 h. The cells were preincubated with different concentrations of FCE/unfermented chickpea milk extract (UCE) and 40 µg/mL GABA for 30 min. The cells were then treated with MnCl_2_ at 37 °C in 5% CO_2_for another 24 h. FCE/UCE was prepared. The samples were centrifuged at 10,000× g for 10 min, and the supernatant was filtered through a 0.22 µm filter. The resulting filtrate was freeze dried. The freeze-dried extract was dissolved in DMEM and diluted to different concentrations.

After 24 h, the mixture was collected. Each well was added with 100 µL of the MTT solution (1 mg/mL) and incubated for another 4 h. Finally, the supernatant was discarded and 150 µL of dimethyl sulfoxide (DMSO) was added into each well to dissolve the formazan. After 10 min, absorbance was determined at 570 nm by using a microplate reader (BioTek Synergy2, Vermont, USA). Cells cultured without FCE/UCE and MnCl_2_ were used as control. Cell viability was calculated as follows: cell viability (%) = (As/Ac) × 100, where As is the absorbance in the presence of the sample and/or MnCl_2_ and Ac is the absorbance of the control in the absence of the sample and MnCl_2_.

#### Observation of morphological changes

PC12 cells cultured normally, treated with 750 µM MnCl_2_ only, and protected with 2,000 µg/mL FCE, 2,000 µg/mL UCE, and 40 µg/mL GABA were observed with an inverted microscope (ECLIPSE TE2000-S, Nikon, Japan).

#### Determination of LDH activity (lactate dehydrogenase)

PC12 cells were exposed to MnCl_2_ in the presence or absence of different concentrations of FCE/UCE or 40 µg/mL of GABA for 24 h. The culture medium was collected, and a commercially available assay kit (Nanjing Jiancheng, China) was used to determine LDH activity according to the manufacturer’s protocol. Absorbance was determined at 450 nm.

### Statistical analysis

Each sample was analyzed in triplicate, and mean values were calculated. Analysis of variance (ANOVA) and Duncan’s multiple comparison tests were used to determine significant differences among means (*P* < 0.05) by using IBM SPSS Statistics.

**Table 2:** Screening of GABA-producing LAB in MRS broth and chickpea milk containing 1% and 0.2 % MSG, respectively.

Strains	MRS with 1% MSG		Chickpea milk containing 0.2 % MSG	
	GABA (mg/L)	pH	GABA (mg/L)	pH
M-9	529.23 ± 4.12b	4.31	252.56 ± 4.12c	4.25
M-6	545.33 ± 5.43a	4.42	282.12 ± 4.03a	4.32
M-7	541.52 ± 4.24a	4.48	265.32 ± 3.01b	4.28
M-5	333.68 ± 4.44c	4.29	205.59 ± 3.03e	4.21
22	232.80 ± 5.28e	4.22	186.34 ± 2.91f	4.01
W-21	240.46 ± 5.56e	4.20	153.71 ± 2.21g	4.05
P-1	270.24 ± 4.67d	4.21	232.27 ± 4.33d	4.18
5	126.46 ± 4.15f	4.15	115.58 ± 2.42h	4.05
M-8	129.26 ± 3.12f	4.14	104.36 ± 3.42j	4.10
E–5	123.24 ± 3.36f	4.16	122.25 ± 2.52i	4.08

**Notes.**

Data are expressed as mean ± SD from triplicate experiments. Different superscripts within the same column indicate significant difference (*P* < 0.05).

## Results and Discussion

### Isolation and screening of GABA-producing LAB

LAB strains isolated from traditional fermented foods, such as *paocai*, were screened for their GABA-producing ability in MRS broth supplemented with 1% MSG. Paper chromatography analysis showed that 17 of the 377 strains produced GABA (representative results are shown in Fig. S1); of these strains, 10 strains produced GABA at concentrations higher than 100 mg/L as confirmed by HPLC analysis (Table 2). Strain M-6 produced the highest GABA content; ca. 545.33 and 282.12 mg/L GABA were detected after 48 h of fermentation with MRS (fortified with 1% MSG) and chickpea milk (fortified with 0.2% MSG), respectively. Chickpea milk fermented by M-6 also had good overall acceptance based on sensory evaluation results (Table S1).

GABA detected by paper chromatography and HPLC analyses were characterized through LC–MS. The molecular weight of the derivative is 279.35 g/mol. Mass spectrometry identification of the OPA derivative of the GABA standard and the culture supernatant of chickpea milk revealed that this compound had an m/z value of 280.2000 [M + H]^+^ (Fig. S2). Thus, strain M-6 was confirmed to convert MSG to GABA in chickpea milk.

### Identification and DNA sequencing of strain M-6

Physiological and biochemical tests were conducted to determine the identity of the M-6 strain. The bacterium was non-spore forming, rod-type, hetero-fermentative, Gram positive, and negative for catalase and motility. M-6 also did not produce gas and ammonia from glucose and arginine. Thus, the strain was identified as *Lactobacillus*. API carbohydrate fermentation pattern testing showed that the strain was *L. plantarum*. The 16S rDNA sequence of the strain M-6 was 1,477 bp, which showed 99% homology with that of *L. plantarum* strains JNB25 and LP-1. The API identification result was confirmed by 16S rDNA sequencing, and the strain was identified as *L. plantarum* M-6. The sequence of *L. plantarum* M-6 strain was deposited in NCBI Genbank under the accession number KU214638.

### Characterization of glutamate decarboxylase (GAD) gene from *L. plantarum* M-6

**Figure 1:**
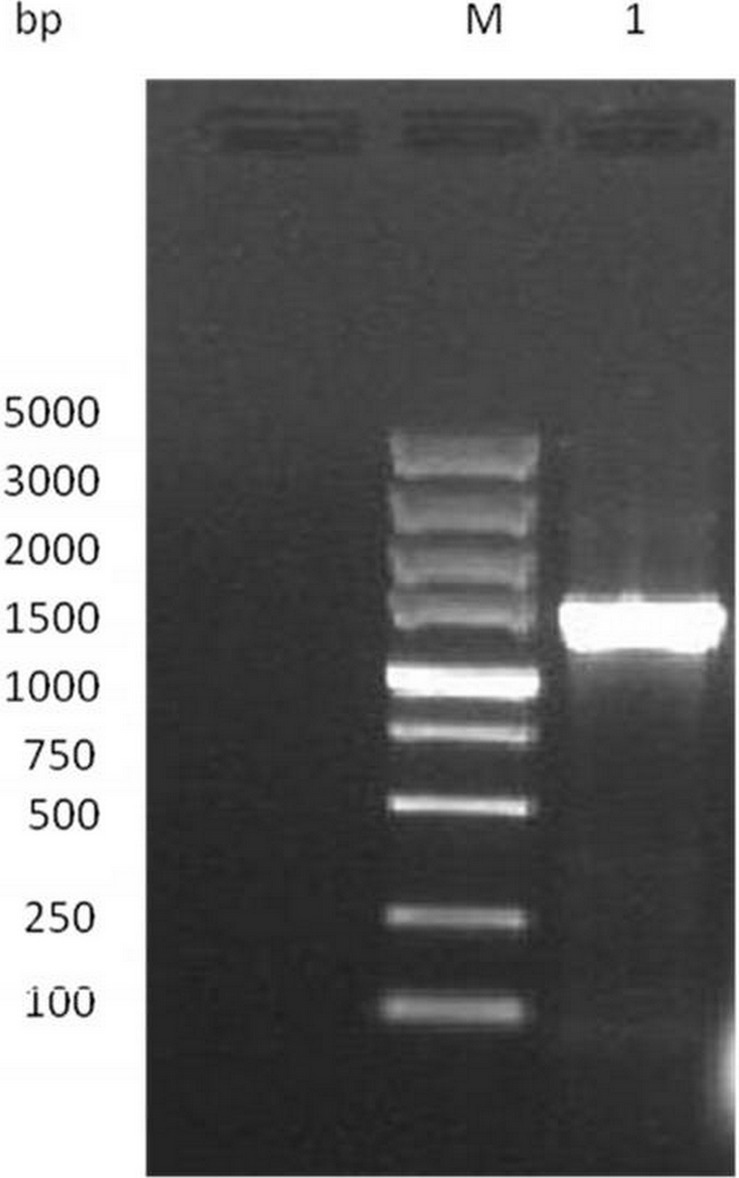
Electrophoresis analysis of GAD gene. Lane M, DL 2000 DNA Marker; lane 1, GAD gene of the strain.

**Figure 2:**
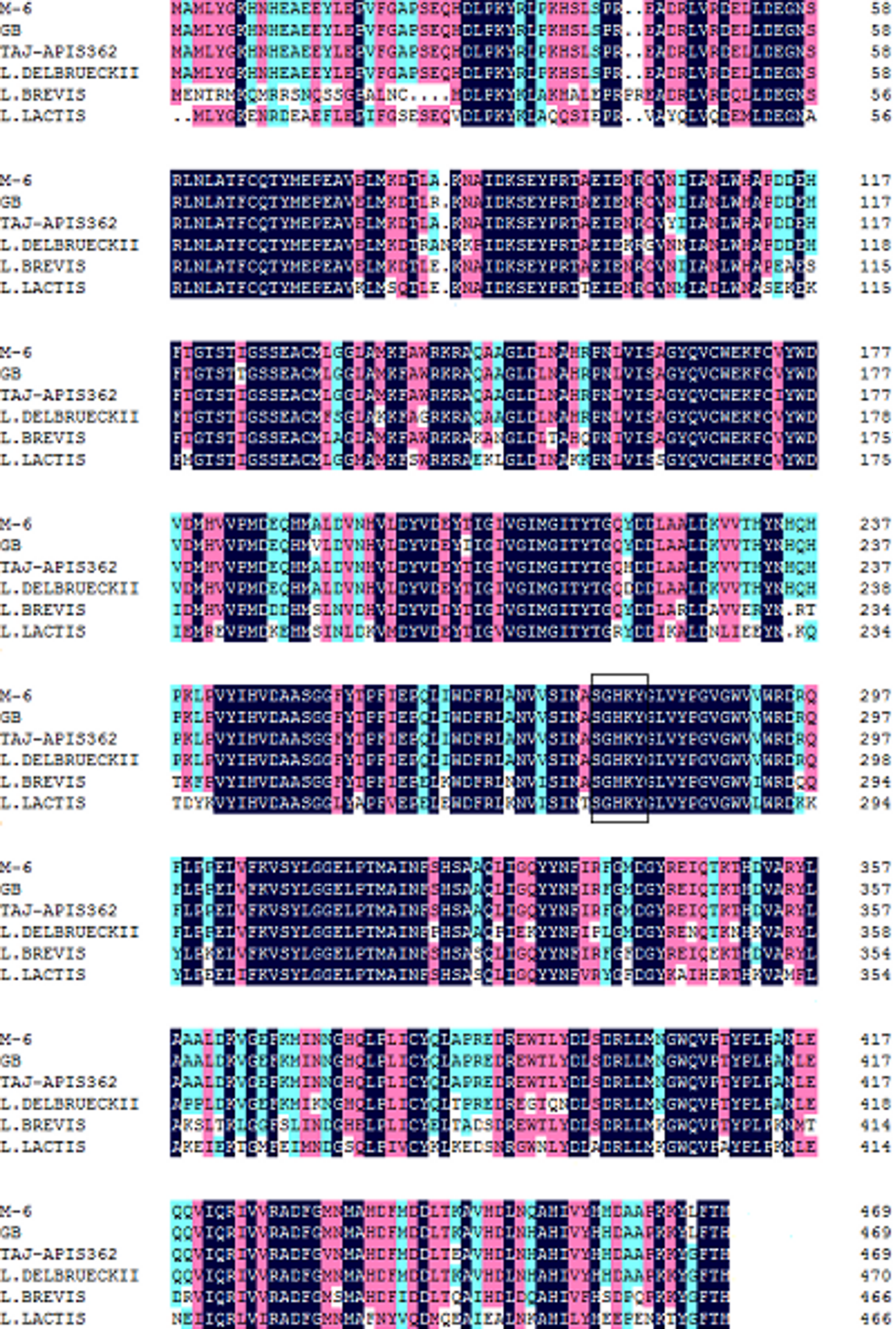
Multiple alignment of GAD from *Lactobacillus plantarum* M-6 and other GAD proteins. Amino acid sequences of GAD from *L. plantarum* M-6 (M-6), *L. plantarum* GB 01-21 (GB, GenBank accession no. AEL29212), *L. plantarum* Taj-Apis362 (Taj-Apis362, AHG59384), *L. delbrueckii subsp. bulgaricus* ATCC 11842 (*L. delbrueckii*, AHX56283), *L. brevis* OPK-3 (*L. brevis*, AAZ95185), and *Lactococcus lactis* (*L. lactis*, WP_023164198) were analyzed using ClustalW (1.81). The box represents the PLP binding site.

The full length of GAD gene from *L. plantarum* M-6 was successfully amplified and sequenced (Fig. 1). The gene was 1,410-bp long, encoding a protein of 469 amino acid residues (Fig. 2) with a predicted molecular weight of 53.7 kDa and pI of 5.58. Similarly, the full-length GAD gene was also amplified from other LAB strains, such as *L. plantarum* Taj-Apis362 (Tajabadi et al., 2015), *L. paracasei* (Komatsuzaki et al., 2008), and *L. brevis* OPK-3 (Park & Oh, 2007). The amino acid sequence of *L. plantarum* M-6-derived GAD showed a similarity of 99%, 99%, 94%, 94%, and 68% with the other GAD proteins from *L. plantarum* GB 01–21, *L. plantarum* Taj-Apis362, *L. delbrueckii* subsp. *bulgaricus* ATCC 11842, *L. brevis* OPK-3, and *Lactococcus lactis,* respectively. Among the analyzed GAD proteins, a similar sequence encoding amino acids SGHKY, including lysine, was found and identified as essential to the binding of PLP (a cofactor of GAD) (Park & Oh, 2007). Thus, GAD gene was confirmed to be present in *L. plantarum* M-6. The GAD gene of *L. plantarum* M-6 was submitted to GenBank under the accession number KU214639.

### Determination of the optimum culture conditions for GABA production in chickpea milk by *L. plantarum* M-6

**Figure 3:**
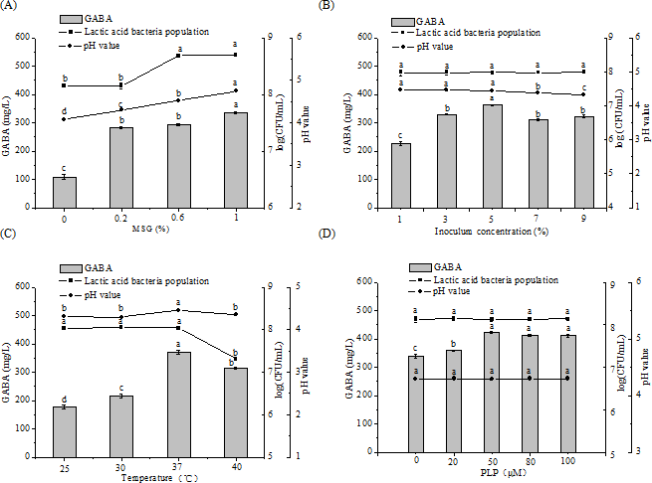
Changes in GABA concentrations, LAB counts, and pH values in chickpea milk fermented for 48 h containing different concentrations of MSG (A), under different inoculum concentrations (B), at different fermentation temperatures (C), and containing different concentrations of PLP (D). Data are expressed as mean ± SD from triplicate experiments. Different letters at the top of the bars indicate significant difference (*P* < 0.05).

Figure 3 shows the effect of MSG concentration, inoculum concentration, culture temperature, and PLP concentration on GABA production, LAB populations, and pH level of fermented chickpea milk. The GABA concentration increased with MSG supplementation at low levels (0%–0.2%); further increase in the MSG concentration to 0.6% did not significantly affect GABA production but resulted in lower conversion yield (9%) than that of 0.2% MSG supplementation (26%). MSG could be a minor nutritional element to promote the growth of M-6. The pH of the fermented chickpea milk increased with increasing MSG concentration possibly because of the presence of alkaline MSG remnants. Considering the results for taste, GABA concentration, and conversion rate, 0.2% MSG was selected as the optimal MSG concentration. Moreover, the yield of GABA initially increased with increasing inoculum concentration (v/v), until the concentration reached 5% and yield started to decrease. pH level decreased when the inoculum concentration was increased from 1% to 5%. Inoculum concentration minimally influenced LAB counts and pH (*P* > 0.05). However, temperature strongly influenced both microbial growth and GABA production. An elevated temperature increased the reaction rate and influenced the final yield of GABA. The optimal temperature for GABA production and LAB growth was 37 °C. Temperatures higher than 37 °C cause enzyme inactivation and cell aging, leading to decrease in GABA production and microbial growth. As a cofactor of the enzyme, PLP plays an important role in stimulating GAD activity (Tong et al., 2002). GABA production reached its highest when PLP concentration increased from 0 µM to 50 µM; higher PLP concentration resulted in no obvious effect on GABA concentration (*P* > 0.05). This trend may be due to that the amount of PLP at 50 µM is enough for promoting GAD activity. Addition of PLP had little effect on LAB growth and pH value (*P* > 0.05), suggesting that PLP cannot be used as a nutritional factor for M-6 growth. The optimal concentration of PLP was established as 50 µM, at which GABA production is increased by 24.48% relative to that of the control (442.39 mg/L).

Based on the results of the single-factor test, chickpea milk fortified with 0.2% MSG was chosen as the culture substrate for GABA production. The three variables applied in BBD were inoculum concentration, temperature, and PLP concentration. Table 1 shows the 17 various combination sets produced and the corresponding GABA yields for both the predicted and actual values. Table 3 presents the regression coefficients and the ANOVA results for BBD. An *F*-value of 54.88 indicated that the model was significant (*P* < 0.0001). The value of the determination coefficient (*R*^2^) was 0.9860, indicating that the experimental data fitted well with the model. ${R}_{\mathrm{adj}}^{2}$ was 0.9895, which is close to *R*^2^, confirming that the model was highly significant. The model also possessed non-significant lack-of-fit values (*F* = 0.14, *P* = 0.9290). The results above proved the validity of the experimental model, which can be used to describe the real relationship between the variables and the yield of GABA. Values of *P* value less than 0.05 indicate that the model terms are significant, so the independent variables, A and B, the quadratic terms of A, B, and C, and the interaction between A and B had significant effects on GABA production.

**Table 3:** Analysis of variance (ANOVA) for the fitted quadratic polynomial model.

Source	Sum of squares	*df*	Mean Square	*F* Value	*P* value	Significance
Model	3.169E+005	9	35211.12	54.88	<0.0001	^**^
A	22941.89	1	22941.89	35.75	0.0006	^**^
B	1.405E+005	1	1.405E+005	219.01	<0.0001	^**^
C	830.28	1	830.28	1.29	0.2928	
AB	3875.69	1	3875.69	6.04	0.0436	^*^
AC	118.37	1	118.37	0.18	0.6805	
BC	464.83	1	464.83	0.72	0.4229	
A^2^	4436.63	1	4436.63	6.91	0.0339	^*^
B^2^	1.243E+005	1	1.243E+005	193.71	<0.0001	^**^
C^2^	11039.04	1	11039.04	17.20	0.0043	^**^
Residual	4491.62	7	641.66			
Lack of fit	435.05	3	145.02	0.14	0.9290	
Pure error	4056.57	4	1014.14			
Cor total	3.214E+005	16				
	*R*^2^ = 0.9860	${R}_{\mathrm{adj}}^{2}=0.9681$				

**Notes.**

* Significant at 0.05 level.

** Significant at 0.01 level.

**Figure 4:**
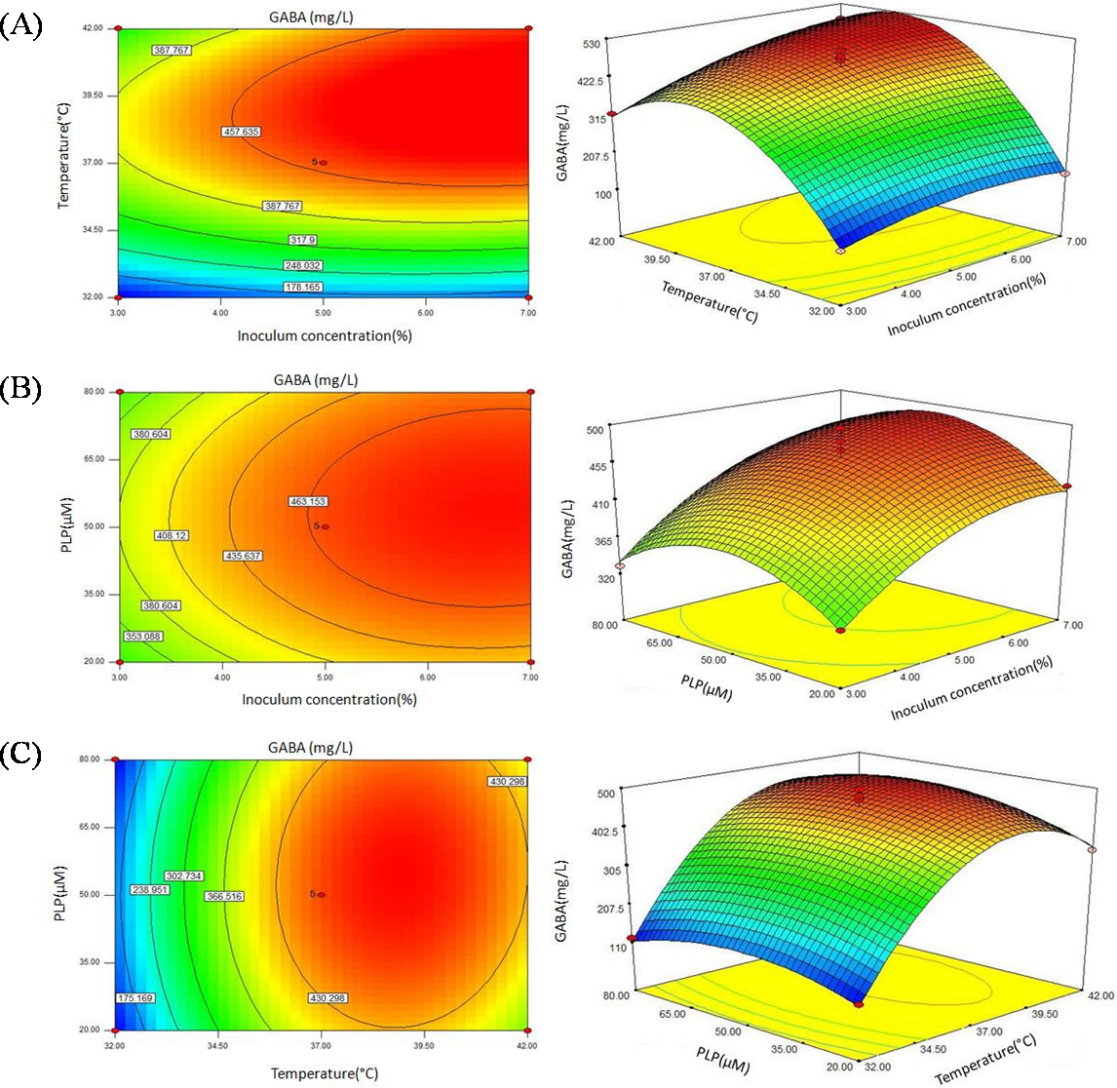
Contour plots and response surface plots showing the effect of inoculum concentration, temperature, and PLP concentration on GABA yield.

The contour and response surface plots in Fig. 4A shows steep surfaces, indicating the effects of inoculum concentration and temperature interaction on GABA production were highly significant, with *P* value of 0.0436. On the contrary, the response surface and contour plots in Figs. 4B and 4C shows smoother surfaces, indicating the effects of inoculum concentration and PLP interaction, and PLP and temperature interaction on GABA production were not significant, with a *P* value of 0.6805 and 0.4229, respectively.

According to RSM results, the optimal culture conditions for GABA production were obtained as follows: inoculum concentration of 7%, culture temperature of 39.42 °C, and PLP concentration of 56.10 µM. Under these conditions, the maximum GABA production was predicted to be 529.71 mg/L. Considering the conditions for actual production, the optimal conditions were modified as follows: inoculum concentration of 7%, culture temperature of 39 °C, and PLP concentration of 55 µM. Under these conditions, a mean value of 527.26 ± 2.53 mg/L (*n* = 3) was obtained from actual experiments, which is close to the predicted value of 529.71 (*P* > 0.05). The results demonstrated that the response model was adequate for the optimization of GABA production.

### Time course synthesis of GABA during fermentation

**Figure 5:**
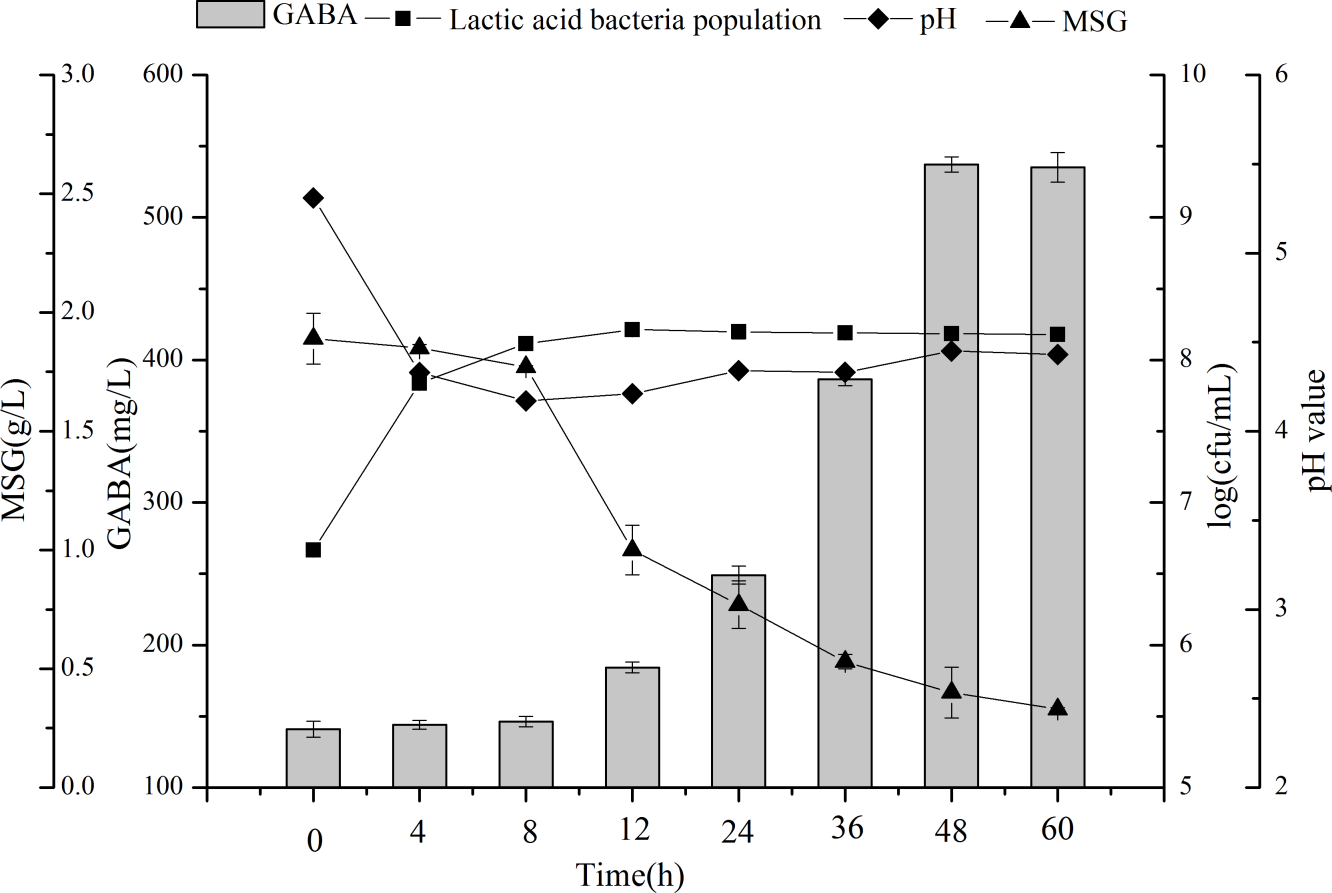
Changes in LAB counts, pH value, GABA, and MSG concentrations during fermentation with *L. plantarum* M-6. Data are expressed as mean ± SD from triplicate experiments. Different letters at the top of the bars indicate significant differences (*P* < 0.05).

Figure 5 shows GABA content, MSG content, LAB count, and pH value at different fermentation time points and under the determined optimal conditions. The strain grew rapidly after inoculation and reached the stationary phase after 8 h of incubation, with a LAB population of 8.11 log (cfu/mL), which is 1.44 log cycles greater than the value obtained at the beginning of incubation. To exert the therapeutic benefits *in vivo*, bacterial populations in probiotic foodstuff reportedly should be at least 10^6^ cfu/mL (Ghosh, Chattoraj & Chattopadhyay, 2013). The LAB count in our study was similar to that obtained from soy milk fermented by *Enterococcus faecium* strains, which had a cell density value ranging from 8.06–8.96 log (cfu/mL) after 16 h of fermentation (Cristina et al., 2012). The value obtained in the current study was slightly higher than that obtained from black soybean milk fermented by *L. brevis* FPA3709, which had a bacterial count of 1.2 × 10^8^ cfu/mL after fermentation at 37 °C for 24 h (Ko, Lin & Tsai, 2013). Thus, chickpea milk is suitable for LAB growth and can be used as a good, functional food carrier.

GABA content increased slowly in the first eight hours of fermentation, then rapidly to reach a maximum of 537.23 mg/L at 48 h. Past that time point GABA production reached a plateau (*P* > 0.05). With the increment of GABA, MSG was consumed, and resulted in a residual concentration of 0.33 g/L. Thus, GABA production does not appear to be growth-associated, and instead occurs in the stationary phase of bacterial growth. GABA was shown to be produced by LAB fermentation from many kinds of legume, including black soybean milk and lentil (Ko, Lin & Tsai, 2013; Torino et al., 2013). To the best of our knowledge, the present study is the first to demonstrate the production of GABA from chickpea milk by LAB fermentation.

The pH level sharply decreased from 5.31 to 4.17 during the first 8 h probably because of the production of organic acids by LAB during fermentation (Schindler et al., 2012). The result is consistent with the LAB counts during the first 8 h, in which *L. plantarum* M-6 had a rapid growth. Subsequently, the pH value increased slightly to 4.45 at 48 h. These results agree with a previous study by Villegas et al. (2016). The probable reason for the increment of pH value follows. First, GAD activity allows the producing bacteria to overcome the low-pH stress of fermented food. When GABA is produced, glutamate is taken in by a specific transporter, then an intracellular proton is removed during glutamate decarboxylation. Meanwhile, via an antiporter, GABA is exported from the cell, thus increasing the cytoplasmic pH, and also slightly increasing the extracellular pH (Cotter & Hil, 2003).

### Neuroprotective effect of FCE on manganese-induced PC12 cell death

#### Effect of FCE on cell viability and cell morphology

**Figure 6:**
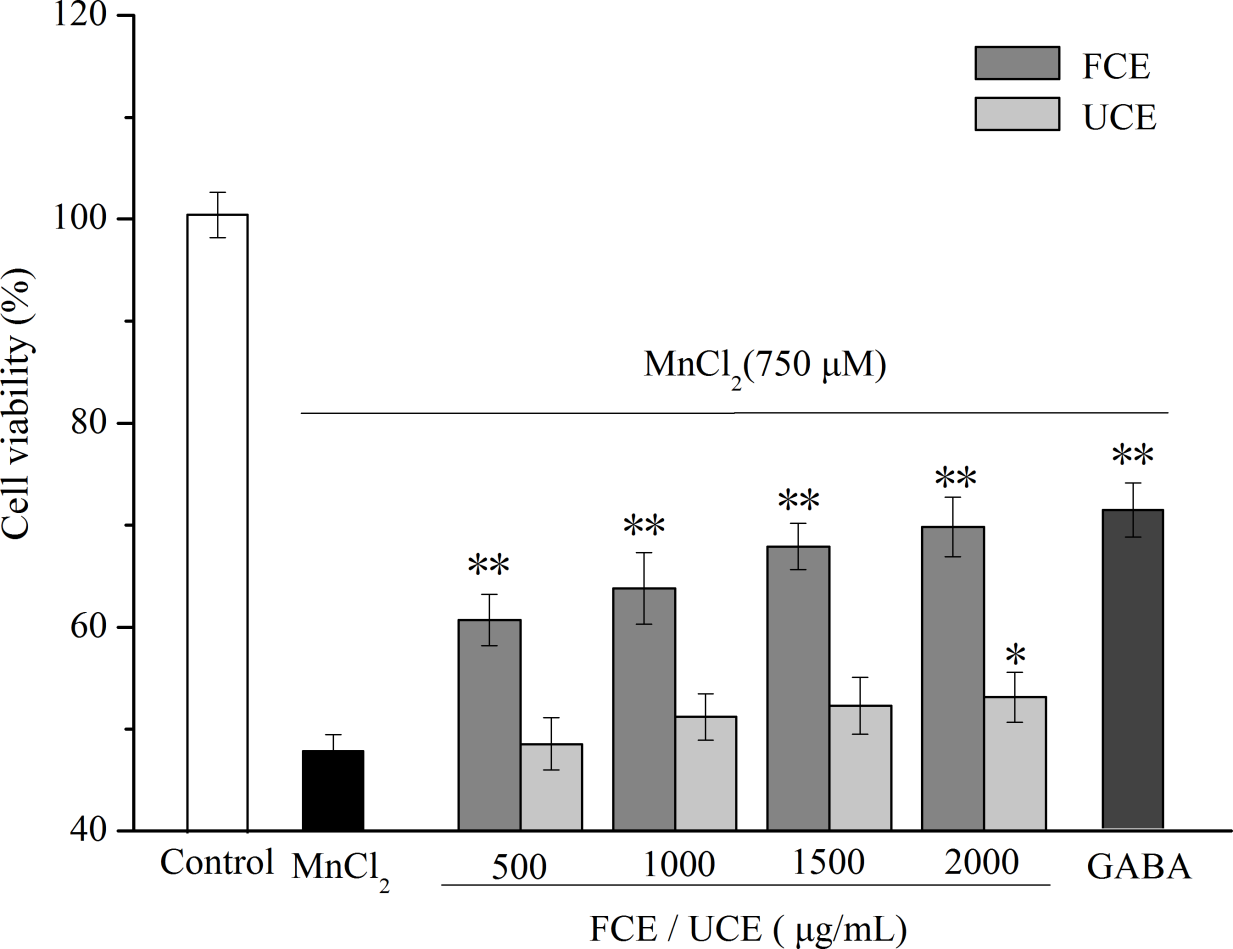
Effect of fermented chickpea milk extracts (FCE) and unfermented chickpea milk extracts (UCE) on manganese-induced PC12 cell death. GABA (40 µg/mL) was used as positive control. All data are reported as mean ± SD (*n* = 3), ^∗^ indicates statistical difference (*P* < 0.05) with MnCl_2_ treated group, ^∗∗^ indicates statistical difference (*P* < 0.01) with MnCl_2_ treated group.

**Figure 7:**
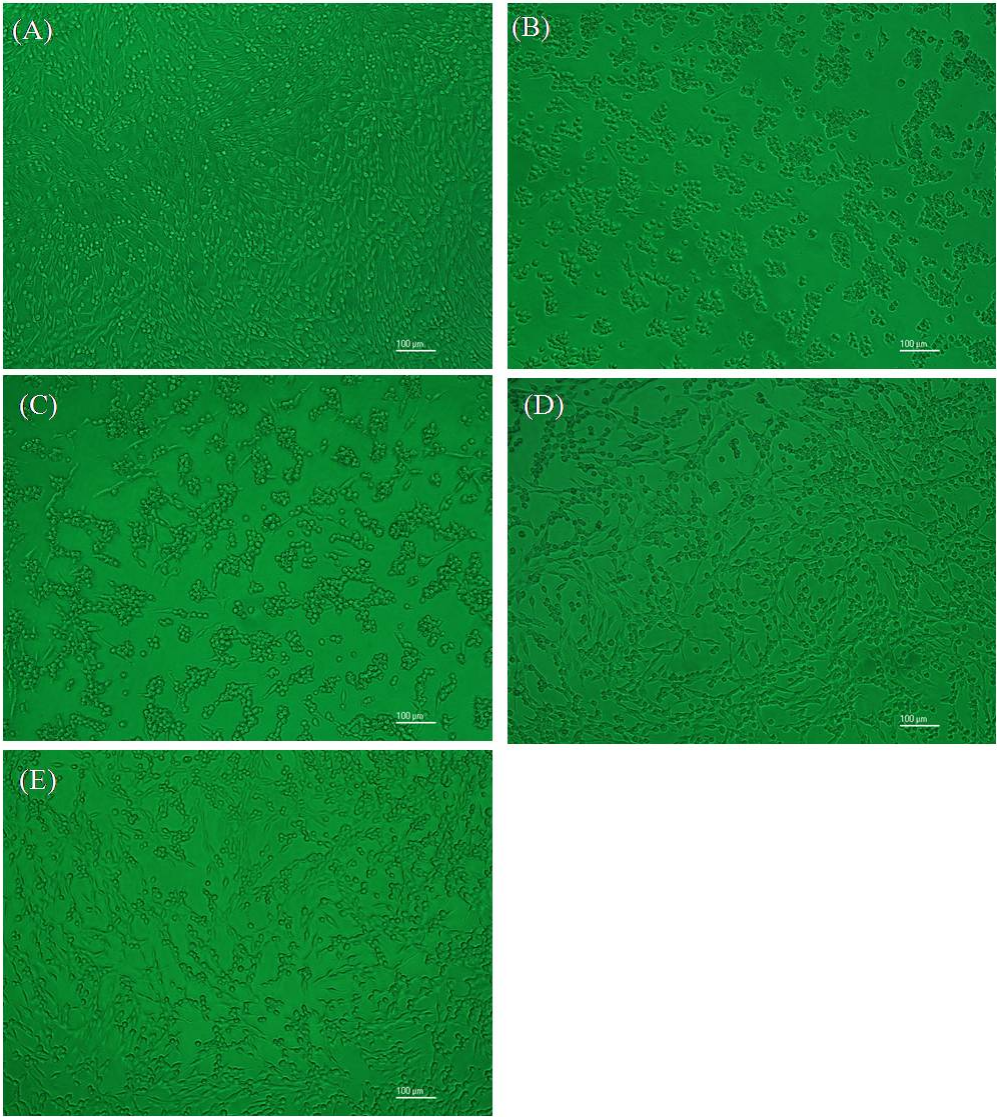
Morphology of PC12 cells observed by inverted microscope. (A) Normal cells without MnCl_2_ or samples, (B) cells injured by MnCl_2_ (750 µM), cells treated with (C) UCE (2,000 µg/mL) + MnCl_2_(750 µM), (D) FCE (2,000 µg/mL) + MnCl_2_ (750 µM), and (E) GABA (40 µg/mL) + MnCl_2_ (750 µM).

The amount of bioactive component in GABA as contained in FCE is 17.78 mg/g. To determine the effect of GABA on cell viability, 40 µg/mL of GABA was chosen as positive control, comparable to the content of GABA in 2,000 µg/mL of FCE. The survival of the PC12 cells was determined by MTT assay. As shown in Fig. 6, MnCl_2_ treatment reduced cell survival. A concentration of 750 µM MnCl_2_ resulted in a cell viability rate of 47.87%. After pretreatment with different concentrations of FCE (500–2,000 µg/mL) or GABA, cell viability significantly increased by 26.80%–48.80%. Thus, FCE protected PC12 cells from injury in a dose-dependent manner, whereas UCE only showed little protective effect at its highest concentration. The morphological changes of PC12 cells under different conditions were also observed. Untreated cells were exuberant and fibriform, showed a distinct boundary and were associated with the cells (Fig. 7A). By contrast, when treated with MnCl_2_, the number of PC12 cells decreased and most of the cells crimpled to a spherical point and assembled; moreover, the links among the cells were absent (Fig. 7B). After pretreatment with UCE, not much changes were apparent (Fig. 7C). In FCE or GABA pretreatment, some PC12 cells regained their inherent morphology, that is, a fibriform structure and a distinct boundary (Figs. 7D–7E).

Manganese at elevated concentrations is toxic and can cause irreversible damage to the central nervous system. Occupational exposure to manganese results in a syndrome that resembles Parkinson’s disease (Pal, Samii & Calne, 1999). PC12, a clonal cell line of rat pheochromocytoma, has similar physiological characteristics to neuronal cells, and is used for neuronal investigations (Jiang et al., 2003). Manganese has also been reported to induce apoptosis in PC12 cells (Hirata, 2002). Thus, PC12 cells can be employed as a model to evaluate the protection of different samples on manganese-induced injury. Based on the results above, FCE, which has an enhanced level of GABA, is confirmed to confer a neuroprotective effect against manganese-induced PC12 cell death, probably because of the higher levels of GABA after fermentation. Results agree with a previous investigation in which treatment of PC12 cells with *L. buchneri* MS strain culture medium, with enhanced levels of GABA, conferred protection from cytotoxic MnCl_2_ of 100–500 µM concentration (Cho, Chang & Chang, 2007).

#### Effect of FCE on LDH activity

**Figure 8:**
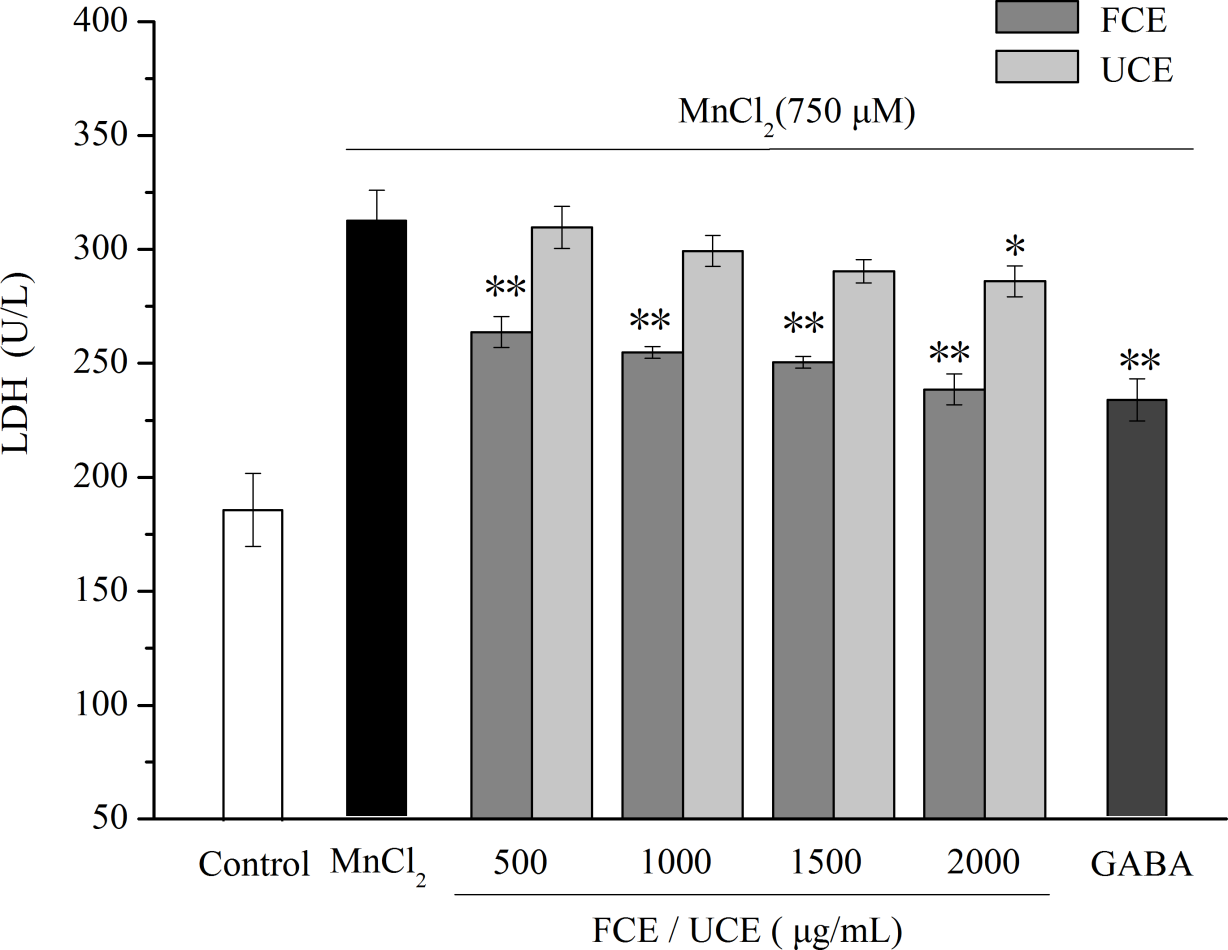
Determination of the LDH activity in PC12 cells. GABA (40 µg/mL) was used as positive control. All data are reported as mean ± SD (*n* = 3), ^∗^ indicates statistical difference (*P* < 0.05) with MnCl_2_ treated group, ^∗∗^ indicates statistical difference (*P* < 0.01) with MnCl_2_ treated group.

When the cell membrane is damaged, LDH is released from inside the cell to the culture medium; thus, LDH activity can be used as an indicator for cell membrane damage. The effect of FCE/UCE on LDH activity in MnCl_2_-treated PC12 cells is shown in Fig. 8. After exposure to MnCl_2_, LDH activity significantly increased in the culture medium of PC12 cells (*P* < 0.01), suggesting serious membrane damage caused by MnCl_2_. FCE or GABA treatment markedly reduced LDH activity in MnCl_2_-treated PC12 cells (*P* < 0.01), whereas UCE showed a slightly reduced LDH activity only at 2,000 µg/mL (*P* < 0.05). The results in LDH activity were in agreement with the morphological assessment and the MTT assay, which all confirm that GABA-enriched FCE can attenuate MnCl_2_-induced injury in PC12 cells. In summary, GABA-enriched FCE protects PC12 cells partially by retaining the integrity of membrane, which is necessary for the viability of cells.

## Conclusion

This study obtained chickpea milk with high GABA content through fermentation by *L. plantarum* M-6, a strain isolated from traditional fermented foods. Optimization by single-factor test and RSM indicated that the fermented chickpea milk contained high amounts of GABA (537.23 mg/L) when fortified with 0.2% MSG and 55 µM PLP and incubated at 39 °C for 48 h. The extract of GABA-enriched fermented chickpea milk demonstrated a neuroprotective effect on manganese-induced PC12 cell death. Thus, the fermented chickpea milk exhibits potential health benefits. Further study is suggested to test whether fermented chickpea milk exerts physiological effect *in vivo*, and to determine the molecular mechanisms underlying the protective effects of FCE against manganese-induced toxicity.

##  Supplemental Information

10.7717/peerj.2292/supp-1 Data S1 Raw data for Figs. 5, 7 and 8 and 10; Tables 1 and 2 and Fig. S2

10.7717/peerj.2292/supp-2 Figure S1 Pre-staining paper chromatography profile of GABA, MSG, and culture supernatant of GABA-producing LAB strains Lane G, GABA standard (2 g/L); lane GM, GABA and MSG standards (2 g/L); lane 1, MRS with 1% MSG (blank control); lanes 2–5, 7–10, 13–15, LAB isolates without capacity for GABA production; lane 6, M-6; lane 11, M-5; lane 12, M-7; lane 16, M-9.

10.7717/peerj.2292/supp-3 Figure S2 MS profiles of the OPA derivatives of GABA standard sample (A), and cultural supernatant of L. plantarum M-6 in chickpea milk containing 0.2% MSG (B)

10.7717/peerj.2292/supp-4 Table S1 Sensory characteristics of fermented chickpea milk Data are expressed as mean ± SD from triplicate experiments. 1 = dislike very much; 2 = dislike; 3 = acceptable; 4 = like; 5 = like very much.

## Additional Information and Declarations

### DNA Deposition

The following information was supplied regarding the deposition of DNA sequences: NCBI Genbank accession numbers KU214638 and KU214639.

### Data Availability

The following information was supplied regarding data availability: The raw data has been supplied as Data S1.
