# Supplementary figures and images for: Novel fermented chickpea milk with enhanced level of γ-aminobutyric acid and neuroprotective effect on PC12 cells
Source: PeerJ. 2016 Aug 4;4:e2292. doi: 10.7717/peerj.2292 (PMC4991855; doi:10.7717/peerj.2292)

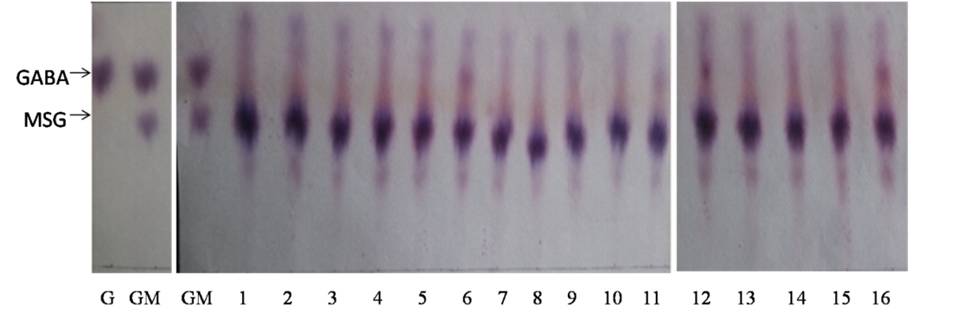

Supplement: Figure S1 — Lane G, GABA standard (2 g/L); lane GM, GABA and MSG standards (2 g/L); lane 1, MRS with 1% MSG (blank control); lanes 2–5, 7–10, 13–15, LAB isolates without capacity for GABA production; lane 6, M-6; lane 11, M-5; lane 12, M-7; lane 16, M-9. [file peerj-04-2292-s002.jpg]

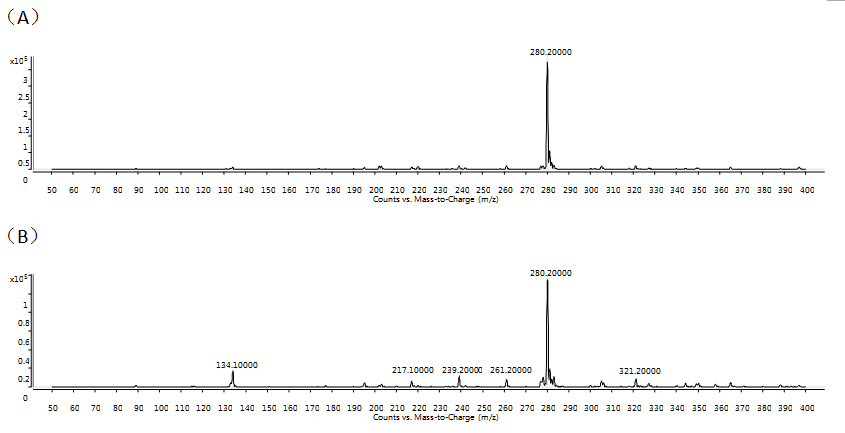

Supplement: Figure S2 [file peerj-04-2292-s003.png]
