# Supplementary material for: Novel fermented chickpea milk with enhanced level of γ-aminobutyric acid and neuroprotective effect on PC12 cells
Source: PeerJ. 2016 Aug 4;4:e2292. doi: 10.7717/peerj.2292 (PMC4991855; doi:10.7717/peerj.2292)
Supplement: Table S1 — Data are expressed as mean ± SD from triplicate experiments. 1 = dislike very much; 2 = dislike; 3 = acceptable; 4 = like; 5 = like very much. [file peerj-04-2292-s004.docx]

 Supplemental Information S2. Sensory characteristics of fermented chickpea milk.

| Items | Scores |
| --- | --- |
| Appearance | 3.54±0.11 |
| Texture | 3.42±0.15 |
| Aroma | 3.46±0.10 |
| Flavor | 3.52±0.10 |
| Taste | 3.76±0.10 |
| Overall acceptability | 3.76±0.05 |

Data are expressed as mean ± SD from triplicate experiments. 1 = dislike very much; 2 = dislike; 3 = acceptable; 4 = like; 5 = like very much.
